# Development of the Relationships Among Dynamic Balance Control, Inter-limb Coordination, and Torso Coordination During Gait in Children Aged 3–10 Years

**DOI:** 10.3389/fnhum.2021.740509

**Published:** 2021-10-28

**Authors:** Hiroki Mani, Saori Miyagishima, Naoki Kozuka, Takahiro Inoue, Naoya Hasegawa, Tadayoshi Asaka

**Affiliations:** ^1^Faculty of Welfare and Health Science, Physical Therapy Courses, Oita University, Oita, Japan; ^2^Division of Rehabilitation, Sapporo Medical University Hospital, Sapporo Medical University, Sapporo, Japan; ^3^Department of Physical Therapy, School of Health Sciences, Sapporo Medical University, Sapporo, Japan; ^4^Graduate School of Health Sciences, Hokkaido University, Sapporo, Japan; ^5^Faculty of Health Sciences, Hokkaido University, Sapporo, Japan

**Keywords:** gait, motor development, balance control, dynamic postural stability, inter-limb coordination, trunk coordination

## Abstract

Knowledge about the developmental process of dynamic balance control comprised of upper arms and upper legs coordination and trunk and pelvis twist coordination is important to advance effective balance assessment for abnormal development. However, the mechanisms of these coordination and stability control during gait in childhood are unknown.This study examined the development of dynamic postural stability, upper arm and upper leg coordination, and trunk and pelvic twist coordination during gait, and investigated the potential mechanisms integrating the central nervous system with inter-limb coordination and trunk and pelvic twist coordination to control extrapolated center of the body mass (XCOM). This study included 77 healthy children aged 3–10 years and 15 young adults. The child cohort was divided into four groups by age: 3–4, 5–6, 7–8, and 9–10 years. Participants walked barefoot at a self-selected walking speed along an 8 m walkway. A three-dimensional motion capture system was used for calculating the XCOM, the spatial margin of stability (MoS), and phase coupling movements of the upper arms, upper legs, trunk, and pelvic segments. MoS in the mediolateral axis was significantly higher in the young adults than in all children groups. Contralateral coordination (ipsilateral upper arm and contralateral upper leg combination) gradually changed to an in-phase pattern with increasing age until age 9 years. Significant correlations of XCOM_ML_ with contralateral coordination and with trunk and pelvic twist coordination (trunk/pelvis coordination) were found. Significant correlations between contralateral coordination and trunk/pelvis coordination were observed only in the 5–6 years and at 7–8 years groups.Dynamic postural stability during gait was not fully mature at age 10. XCOM control is associated with the development of contralateral coordination and trunk and pelvic twist coordination. The closer to in-phase pattern of contralateral upper limb coordination improved the XCOM fluctuations. Conversely, the out-of-phase pattern (about 90 degrees) of the trunk/pelvis coordination increased theXCOM fluctuation. Additionally, a different control strategy was used among children 3–8 years of age and individuals over 9 years of age, which suggests that 3–4-year-old children showed a disorderly coordination strategy between limb swing and torso movement, and in children 5–8 years of age, limb swing depended on trunk/pelvis coordination.

## Introduction

Knowledge of the development process in motor and postural control during gait is a prerequisite for assessing abnormal and pathological development (Sutherland, 1997). However, both growth and central nervous system maturation influence the changes in gait function; thus, the development of gait is a complex matter. Mature development of the mechanisms of inter-limb coordination and dynamic balance control during gait remains unclear.

Previous studies have reported that the fundamental gait pattern matures earlier compared to dynamic balance control and inter-limb coordination patterns during gait. Kinematic and kinetic gait patterns change rapidly with increasing age. Step time-distance parameters (step length, step frequency, walking velocity, and step time) increase with age, whereas cadence is reduced with age (Lythgo et al., 2011; Froehle et al., 2013; Thevenon et al., 2015). These normalized parameters change until approximately 4 years of age (Sutherland, 1997). In addition, kinematics and kinetics (isolated hip, knee, and ankle joint movement) also demonstrate an adult-like pattern by 5 years of age (Vaughan et al., 2003; Ganley and Powers, 2005; Chester et al., 2006). Gait symmetry also improves until 3–4 years of age (Bosch and Rosenbaum, 2010; Lythgo et al., 2011). Therefore, the research suggests that children at about age 5 years have already mastered the basic principle of gait pattern (Hu et al., 2016). Conversely, the process of improving dynamic balance control extends beyond 12 years of age (Meyns et al., 2013, 2020; Hallemans et al., 2018). Development of center of body mass (COM) displacement during gait is a gradual process, which evolves until 7 years of age (Dierick et al., 2004). Distance between center of pressure and COM from double-leg stance to single-leg stance during a single-leg standing task was significantly higher in the 3–10 age group when compared with that in the adult group (Mani et al., 2019). Furthermore, Hallemans et al. (2018) also indicated that spatial margin of stability (MoS) along the mediolateral axis has a strong negative correlation with ages 1–11 years, which is linked to changes in step time-distance parameters of gait. The MoS is an index of dynamic postural stability and is defined as the minimum distance between the boundaries of base of support (BOS) and extrapolated center of body mass (XCOM; Hof et al., 2005). XCOM is defined as the projection on the ground from the COM augmented by a quantity proportional to its velocity (Hof et al., 2005). Thus, it suggests that the development of the dynamic balance control takes longer to mature compared to that of isolated kinematic and kinetic patterns.

Recently, an ability to control inter-limb coordination was also associated with postural stability in pathologic and non-pathologic child populations (Sidiropoulos et al., 2021). However, the developmental processes of the relationships between these factors have not been investigated extensively. Meyns et al. (2013) reported that inter-limb coordination (upper arm and upper leg) becomes gradually effective from 5 years to 12 years of age. They also reported that contralateral limb coordination (left upper arm and right upper leg) takes longer to mature than ipsilateral limb coordination (left upper arm and left upper leg), and children achieve adult-like levels at approximately 14 years of age (Meyns et al., 2020). The contralateral limb coordination pattern gradually approaches the in-phase pattern with increasing age (Meyns et al., 2020). Thus, it is possible that the developmental process of dynamic balance control is associated with inter-limb coordination. Additionally, the trunk and the pelvis represent more than half of the body mass and affect the balance and stability of gait tremendously (Shih et al., 2021). Furthermore, appropriate arm swing and posture of the arm and/or lower limb movement are linked with the development of trunk axial twist coordination (Bruijn et al., 2008; Delabastita et al., 2016; Kiernan, 2021). To the best of our knowledge, only two studies addressed the developmental process of the thorax and pelvic axial twist movement during gait (Thummerer et al., 2012; Li et al., 2021). Thorax and pelvic movement gradually decrease with increasing age from 0 to 16 years (Thummerer et al., 2012; Li et al., 2021), but the development of the relationships between inter-limb coordination and trunk and pelvic twist coordination during gait have not been investigated. A previous study that focused on the coordination of limb and trunk movement reported that children under the age of 5 years showed uncoupled movements of the head, arm, and trunk during a reaching task, and these coupling movements gradually become effective with age (Sveistrup et al., 2008). Furthermore, children from 3 to 8 years depend on “en bloc” postural strategy, defined as having a higher correlation of and limited head, arm, and trunk movements (Assaiante, 1998). Thus, it must be possible that coordination strategy, defined as relationships between inter-limb coordination and trunk and pelvic twist coordination, changes during the growth stages. Knowledge regarding the developmental process of dynamic balance control and inter-limb coordination, and the mechanisms of gait control during childhood are very important to advance effective balance assessment for abnormal development.

We aimed to investigate the development of dynamic balance control with inter-limb coordination and trunk and pelvic twist coordination during gait and the potential mechanisms integrating the central nervous system (CNS) with inter-limb coordination and trunk and pelvic twist coordination to control dynamic balance control. We made the following hypotheses: (1) dynamic postural stability, defined as MoS, gradually improves with increasing age, but is not fully mature at age 10; (2) contralateral limb coordination also gradually approaches the in-phase pattern with increasing age, and is associated with decreasing XCOM fluctuation; (3) trunk and pelvic twist coordination gradually improves with increasing age, and is also associated with improving dynamic postural stability; and, (4) trunk twist coordination contributes to arm swing in children aged 3–8 years. A significant correlation with trunk twist coordination and contralateral limb coordination is present in those 3–8 years old based on previous studies revealing that children depend upon “en bloc” postural strategy until 8 years of age.

## Materials and Methods

### Participants

Seventy-seven healthy children (43 boys and 34 girls) aged 3–10 years and 15 young healthy adults (22.7 ± 2.5 years) participated in the experiment (Table 1). The child population was clustered by age into the following groups: 3–4 years (*n* = 24), 5–6 years (*n* = 26), 7–8 years (*n* = 15), and 9–10 years (*n* = 12). Children who were born after 37 gestational weeks and had a birth weight >2,500 g were recruited. All participants had no significant history of medical, psychiatric, or neurological illness.

**Table 1:** Demographic data.

	3–4 years	5–6 years	7–8 years	9–10 years	Adults
	(*n* = 24)	(*n* = 26)	(*n* = 15)	(*n* = 12)	(*n* = 15)
Sex	Boy 13	Boy 14	Boy 9	Boy 7	Male 7
	Girl 11	Girl 12	Girl 6	Girl 5	Female 8
Age (years)	3.6 ± 0.5	5.5 ± 0.5	7.4 ± 0.5	9.2 ± 0.4	22.7 ± 2.5
Height (cm)	101.3 ± 8.2	113.3 ± 5.9	124.9 ± 4.4	133.5 ± 6.9	167.4 ± 7.1
Body mass (kg)	16.2 ± 2.8	20.4 ± 3.4	23.7 ± 1.6	27.7 ± 3.7	59.5 ± 7.9

Young adults and the parents of each child gave their informed consent prior to the start of the experiment. Furthermore, all children gave their informed assent after this study was explained to them in lay terms prior to the start of the experiment. All study protocols were approved by the ethics committee at the institution where this study took place (28-2-52, F200016), and the experiment was conducted according to the principles of the Declaration of Helsinki.

### Equipment and Procedures

Kinematic data were collected using a VICON Nexus 3D motion-capture system with 10 cameras running at 100 Hz (VICON, Culver City, CA, USA). Twenty-seven reflective markers (9.5 mm in diameter) were placed on the skin or the underwear at bony landmarks: one marker at the vertex, 7th cervical spine, and manubrium, and two markers at the external acoustic foramen, acromioclavicular joint, lateral epicondyle of the upper arm, wrist, head of the third metacarpal, anterior superior iliac spine, posterior iliac spine, lateral epicondyle of the femur, lateral malleolus, second metatarsal head, and calcaneus (Mani et al., 2019). These markers were used for calculating the angle joint movements and the COM with a 14-segment model according to Jensen’s anthropometric data (Jensen, 1986).

**Figure 1:**
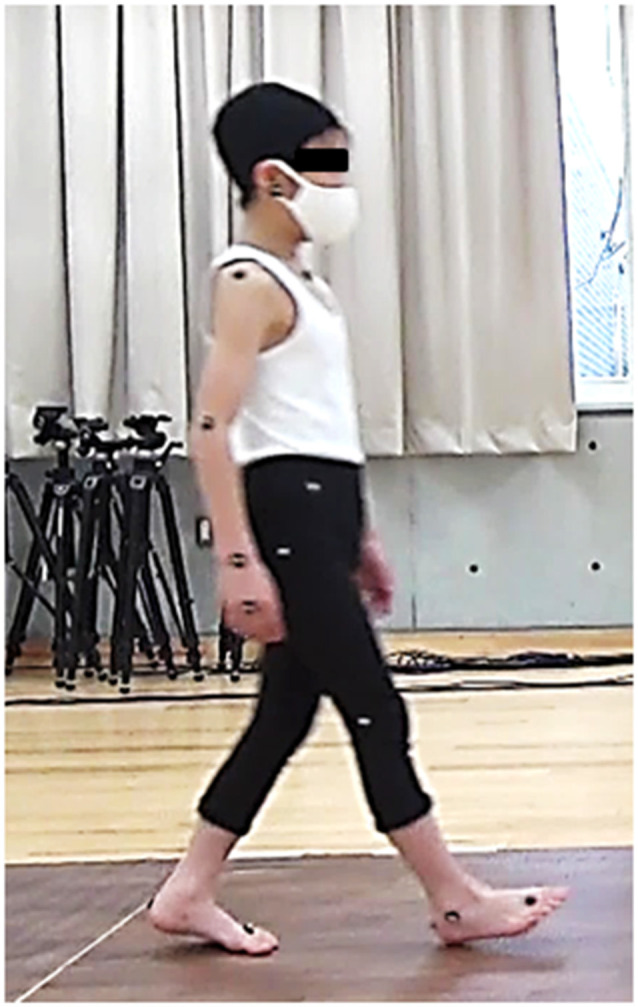
Experimental setup: The participants walk barefoot at a self-selected walking speed along an 8 m walkway. Twenty-seven reflective markers are attached to bony landmarks.

Participants walked barefoot at a self-selected walking speed along an 8 m walkway (Figure 1). Several practice trials were performed before data collection, and each participant was asked to perform five trials with a 2-min rest after each trial.

### Data Analysis

All signals were processed offline using MATLAB R2020b software (MathWorks, Natick, MA, USA). Data from the VICON system were filtered with a 10-Hz fourth-order, zero-lag Butterworth filter.

**Figure 2:**
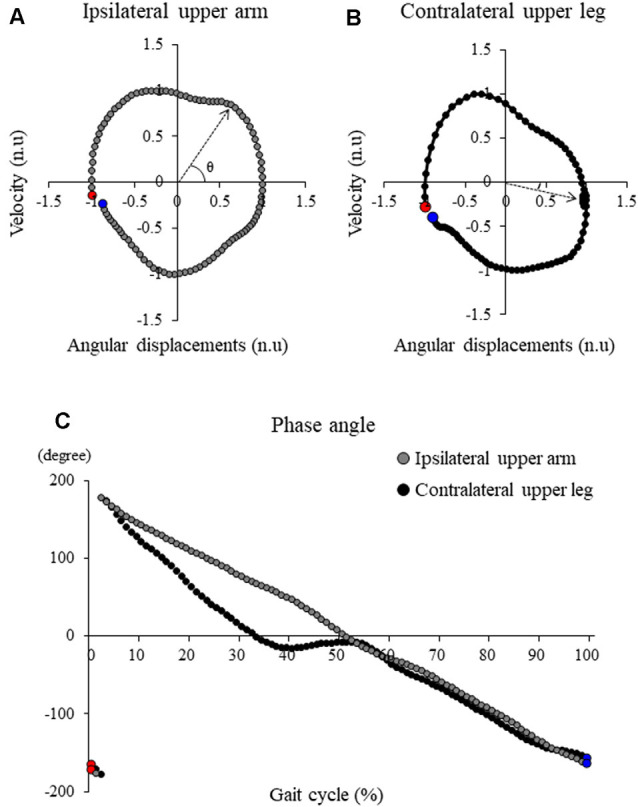
Typical samples of the angle-velocity phase plot in **(A)** ipsilateral upper arm and **(B)** contralateral upper leg and **(C)** the phase angle profile of these segments. Red markers and blue markers represent the first point and the last point during one gait cycle, respectively. Continuous relative phase (CRP) between these segments was calculated by subtracting the specific segment phase angle time series from each other.

Sagittal plane angular displacement and velocity of the upper arm and upper leg segments with respect to the vertical axis (positive value indicates that the distal ends move further from the proximal ends of the segment) were determined (Meyns et al., 2013, 2020). The transverse plane angular displacement and velocity of the trunk and pelvis with respect to the mediolateral axis (positive value indicates that the ipsilateral side moves further from the contralateral side of the segment) were also determined. Trunk axial rotation angle was defined as the angle between the mediolateral axis and a line along both acromioclavicular joints in a transverse plane. Pelvic axial rotation angle was also defined as the angle between the mediolateral axis and a line along both anterior superior iliac spines in a transverse plane. Each maximum absolute angular displacement and velocity during one gait cycle were calculated. One gait cycle was determined as the duration from the first foot contact (FC) on the ipsilateral side to the next FC on the same side. FC was defined as the time at which the heel marker of the swing leg in the vertical direction reached the lowest height. Then, these displacement and velocity measurements were normalized in time (in the percentage of stride duration) and in amplitude (minimum −1 and maximum 1; Meyns et al., 2013, 2020). From these normalized values, the phase plots were determined (normalized angular velocity with respect to normalized angular displacement; Figures 2A,B). Phase angles of each segment were then determined as arctangent from each phase plot (Meyns et al., 2020; Figure 2C). Subsequently, the continuous relative phase (CRP) between different segments was calculated by subtracting the specific segment phase angle time series from each other. The absolute CRP was calculated between 0° and 180°; 0° indicated two segments were moving in the same direction at the same time (in-phase); 180° indicated the segments were moving in opposite directions at the same time (anti-phase). Next, the mean CRP over the gait cycle was referred to as the mean relative phase (MRP). Since there are different phase coupling movements of segments between which coordination was measured, both upper arm combination (both arm combination), both upper leg combination (both leg combination), ipsilateral upper arm and ipsilateral upper leg combination (ipsilateral combination), ipsilateral upper arm and contralateral upper leg combination (contralateral combination), trunk and pelvic combination (trunk/pelvis combination) were calculated.

The MoS and the XCOM were calculated to evaluate the dynamic postural stability and balance control in the frontal plane (Hof et al., 2005). XCOM is calculated by


$$XCOM\;=\;x\;+\;\frac{v}{\sqrt{g/l}}$$

where *x* is the COM displacement, *v* is the COM velocity, *g* is the acceleration of gravity, and *l* is the distance from the axis of ankle joint to the COM position in the frontal plane. Peak XCOM in the mediolateral axis during each gait cycle was also calculated (XCOM_ML_). MoS along the mediolateral axis (MoS_ML_) is defined as the minimum distance from XCOM to the boundaries of the BOS and is calculated by


MoS = boundaries of BOS − XCOM

The boundaries of BOS were defined as the mediolateral position of the ankle marker in the stance leg. XCOM_ML_ was normalized by the distance from the floor to the vertical COM position during static standing (% COM height). The COM velocity was also calculated by dividing the total path length of COM in the anteroposterior axis by the duration of the gait cycle to assess the walking speed. The COM velocity was normalized by *√gl* (Hof, 1996).

### Statistical Analyses

A priori power analysis was performed in G*power 3.1. The sample size was estimated from a pilot study carried out on 25 participants (five participants per group) for a calculated effect size of *f* = 0.466. We performed the power analysis using the F-test model of G*Power 3.1. Twelve participants in each group were deemed sufficient to detect significant differences in the XCOM_ML_ between groups with a power (1-β) of 0.8. Two-way analysis of variance was performed with the factors Group (3–4 years, 5–6 years, 7–8 years, 9–10 years and young adults) and Sex (males and females). If no significant interaction and factorial effect of sex was noted, data pertaining to patients of both sex were combined.

One-way analysis of variance was used to analyze the parameters among the groups (3–4 years, 5–6 years, 7–8 years, 9–10 years, and young adults). The Tukey-Kramer *post hoc* analysis was performed when appropriate. Additionally, a nonlinear regression analysis using exponential functions was applied to examine the relationships between XCOM_ML_ and age. Furthermore, Pearson’s correlation coefficient with whole subject data was used to examine the relationships between XCOM_ML_ and each combination to assess the contribution of inter-limb coordination and torso coordination for dynamic balance control. Finally, Pearson’s correlation coefficient was also used to examine the relationships between MRP of the contralateral combination and that of trunk/pelvis combination in each group to assess balance control strategies of the age groups. All statistical analyses were performed using IBM SPSS Statistics version 26 (IBM Corp., Armonk, NY, USA). Statistical significance was accepted at *p* < 0.05. Data are expressed as mean [standard deviation (SD)].

## Results

**Figure 3:**
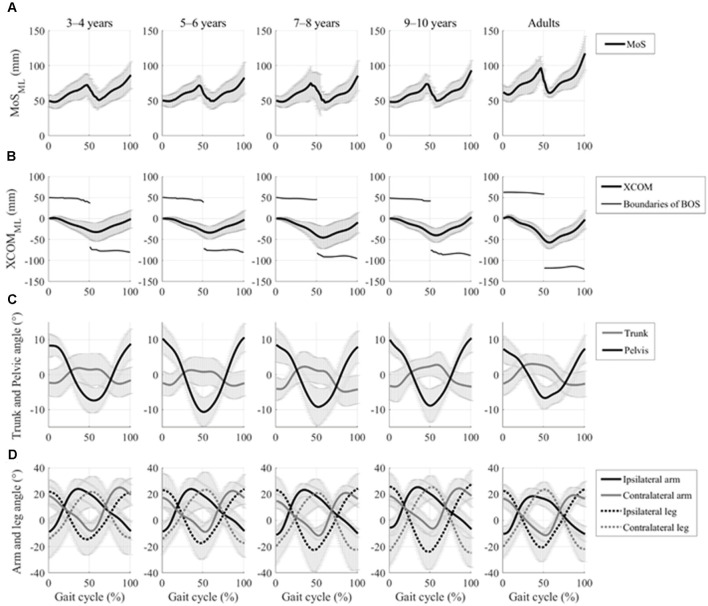
Time profiles of the gait cycle for grand mean spatial margin of stability (MoS), extrapolated center of body mass (XCOM) displacements in the mediolateral axis, and kinematic joint movements with the standard deviation of each group. **(A)** Grand mean mediolateral margin of stability (MOS_ML_) and **(B)** grand mean mediolateral XCOM displacements (XCOM_ML_) and boundaries of base of support (BOS), which is defined by ankle marker on the stance side, and are represented by a thick line and thin line, respectively. **(C)** Grand mean trunk and pelvic rotational angular movements in the horizontal plane are represented by the gray line and the black line, respectively. **(D)** The grand mean of both the upper arm and upper leg joint angular movement in the sagittal plane is represented by the solid lines and the dotted lines, respectively. Ipsilateral and contralateral limbs are represented by the black lines and the gray lines, respectively.

All 92 participants were included in the analyses. Figure 3 shows the time profiles of a gait cycle for the grand mean MoS_ML_, XCOM_ML_, and joint angular movements. Patterns of these data were very similar across all groups, but all the children’s age groups showed more fluctuated patterns in XCOM_ML_ and lower MoS_ML_.

The two-way ANOVA of MOS_ML_, XCOM_ML_, and each coordination showed no significant effect of sex and two-factor interaction (Table 2). Hence, data from both sex were combined.

**Table 2:** Results of two-way analysis of variance for dynamic balance and each coordination.

	Fixed factor	*F* value	*p* value
MOS_ML_	Group	**8.944**	**<0.001**
	Sex	0.707	0.403
	Interaction	0.786	0.537
XCOM_ML_	Group	**4.435**	**0.003**
	Sex	1.182	0.280
	Interaction	1.388	0.245
Both arm combination	Group	**5.820**	**<0.001**
	Sex	0.930	0.338
	Interaction	0.865	0.489
Both leg combination	Group	1.279	0.285
	Sex	3.994	0.050
	Interaction	1.054	0.385
Ipsilateral combination	Group	**2.862**	**0.028**
	Sex	0.722	0.398
	Interaction	0.175	0.951
Contralateral combination	Group	**7.919**	**<0.001**
	Sex	1.156	0.285
	Interaction	0.458	0.766
Trunk/pelvis combination	Group	1.468	0.220
	Sex	<0.001	0.984
	Interaction	0.813	0.520

*Bold denotes significant effects at *p* < 0.05*.

No significant between-group difference was found in the walking speed (*F*_4, 88_ = 0.738, *p* = 0.569; Table 3). Although no significant between-group differences were found in the upper arm and the upper leg angular movements over a gait cycle (*F*_4, 88_ = 0.804, *p* = 0.526 and *F*_4, 88_ = 1.824, *p* = 0.131, respectively; Table 3), significant differences in the trunk and the pelvic angular movements were found between the groups (*F*_4, 88_ = 3.593, *p* = 0.009 and *F*_4, 88_ = 3.817, *p* = 0.007, respectively). A *post hoc* analysis revealed that the trunk angular displacement was significantly decreased in the adult group compared to the 3–4, 5–6, and 7–8 years age groups (*p* = 0.019, *p* = 0.014, *p* = 0.015, respectively), and the pelvic angular displacement was significantly decreased in the young adult group compared to the 5–6 years age group (*p* = 0.002).

**Table 3:** Results of walking speed and the range of motion in each segment over one cycle.

	3–4 years	5–6 years	7–8 years	9–10 years	Adults
	(*n* = 23)	(*n* = 26)	(*n* = 15)	(*n* = 12)	(*n* = 14)
walking speed [n.u]	0.33 ± 0.06	0.35 ± 0.05	0.34 ± 0.05	0.33 ± 0.04	0.32 ± 0.03
Upper arm angular range of motion [°]	42.9 ± 18.3	45.5 ± 18.9	50.6 ± 27.5	54.1 ± 22.2	45.5 ± 13.0
Upper leg angular range of motion [°]	35.2 ± 4.7	34.0 ± 6.2	34.6 ± 5.4	32.3 ± 4.5	31.1 ± 4.5
Trunk angular range of motion [°]	11.7 ± 4.1*	11.8 ± 3.6*	12.3 ± 5.3*	10.7 ± 5.7	7.4 ± 2.4
Pelvic angular range of motion [°]	20.2 ± 5.7	24.1 ± 7.1*	20.6 ± 8.5	21.1 ± 9.2	15.4 ± 4.1

Mean ± SD. Bold denotes significant data. *: *p* < 0.05, compared to that of the adults group.

**Figure 4:**
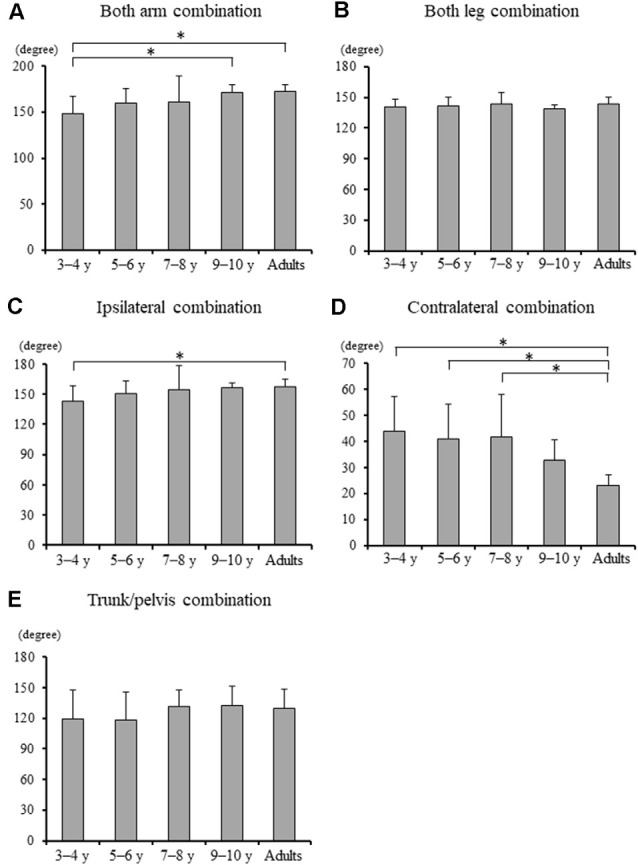
**(A)** Mean relative phase of both upper arm combination, **(B)** both upper leg combination, **(C)** ipsilateral upper arm and leg combination, **(D)** contralateral upper arm and leg combination, and **(E)** trunk and pelvis combination for each group [± standard deviation (SD)]. *, Significant differences *p* < 0.05.

Significant differences in the MRP of both arm combination, ipsilateral combination, and contralateral combination were found between the groups (*F*_4, 88_ = 5.903, *p* < 0.001, *F*_4, 88_ = 3.097, *p* = 0.020, and *F*_4, 88_ = 8.363, *p* < 0.001, respectively; Figure 4). A *post hoc* analysis revealed that the MRP of both upper arm combination was significantly increased in the 9–10 years age and young adult groups than in the 3–4 years age group (*p* = 0.003, *p* = 0.001, respectively), and the ipsilateral combination was significantly increased in the young adult group compared to the 3–4 years age group (*p* = 0.030). Conversely, the MRP of the contralateral combination was significantly decreased in the young adult group compared to the 3–4, 5–6, and 7–8 years age groups (*p* < 0.001). No significant between-group differences were found in the MRP of both leg combination and trunk/pelvis combination (*F*_4, 88_ = 0.924, *p* = 0.454 and *F*_4, 88_ = 1.509, *p* = 0.207, respectively).

**Figure 5:**
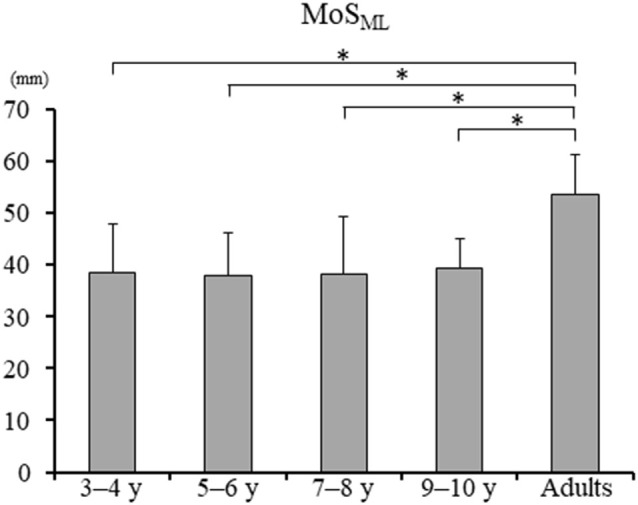
Mean margin of stability in mediolateral axis [MoS_ML_; ± standard deviation (SD)]. *, Significant differences *p* < 0.05.

**Figure 6:**
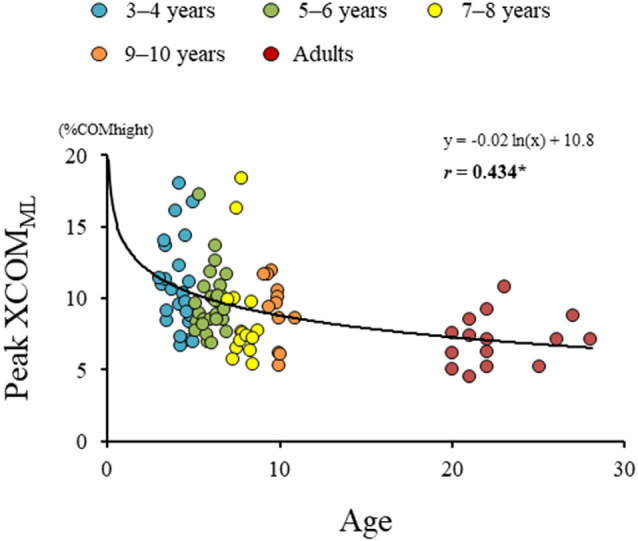
Results of regression analysis performed for age and extrapolated center of body mass in mediolateral axis (XCOM_ML_). *,Significant correlation with age, *p* < 0.05.

**Figure 7:**
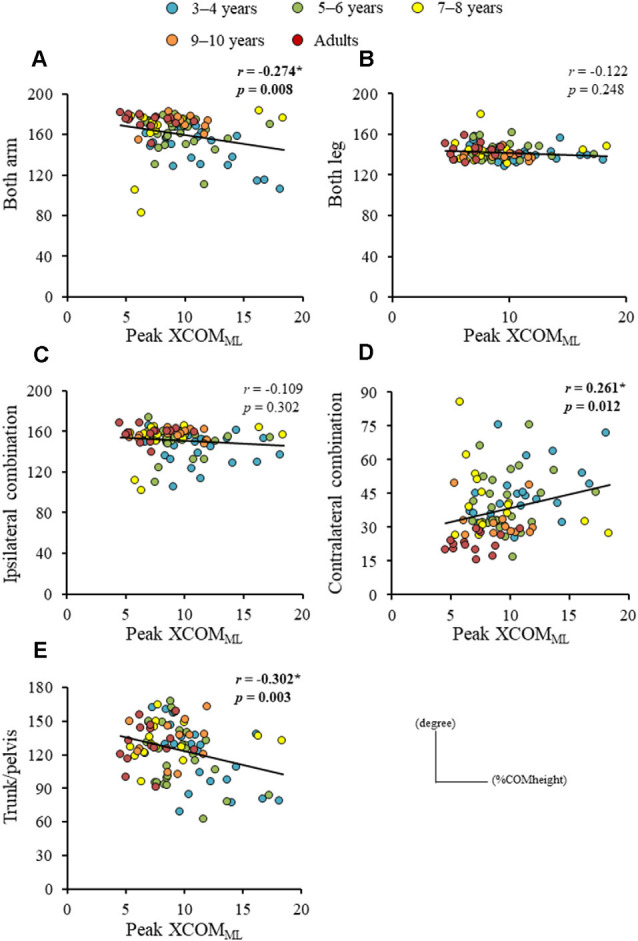
Relationships between peak extrapolated center of body mass (XCOM) displacements in mediolateral axis (XCOM_ML_) and **(A)** mean relative phase (MRP) of both arm combination, **(B)** MRP of both leg combination, **(C)** MRP of ipsilateral arm and leg combination, **(D)** MRP of contralateral arm and leg combination, and **(E)** MRP of trunk/pelvis combination. *,Significant correlation, *p* < 0.05.

**Figure 8:**
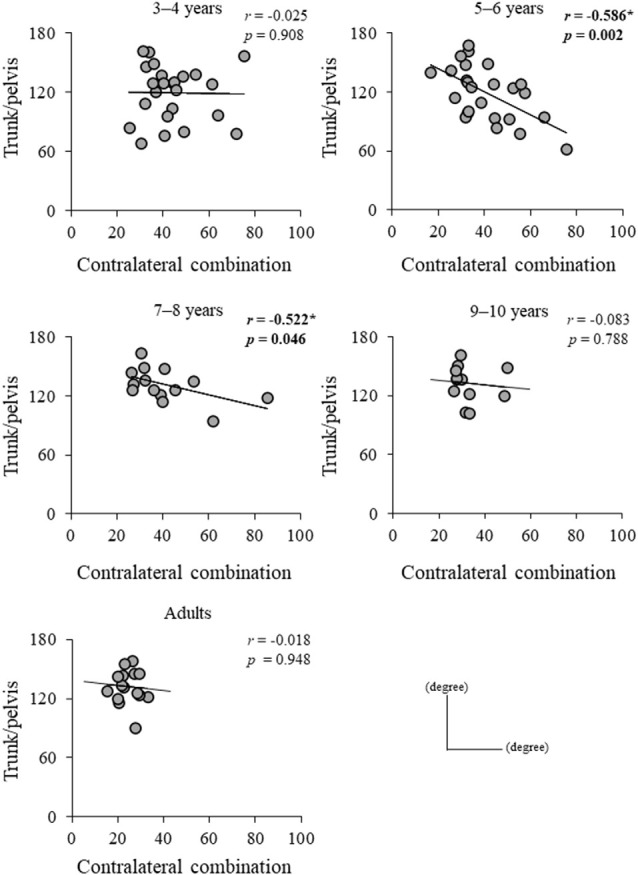
Relationship between mean relative phase (MRP) of contralateral arm and leg combination and MRP of trunk/pelvis combination for each group. *,Significant correlation, *p* < 0.05.

Significant difference in the MoS_ML_ was found between the groups (*F*_4, 88_ = 9.438, *p* < 0.001; Figure 5). A *post hoc* analysis revealed that the MoS_ML_ was significantly higher in the young adult groups than in all children groups (*p* < 0.001, respectively). Peak XCOM_ML_ showed a significant correlation with age (*r* = 0.434, *p* < 0.001; Figure 6). Significant correlations between XCOM_ML_ and MRP of both arm coordination, and between XCOM_ML_ and MRP of contralateral combination, and between XCOM_ML_ and MRP of trunk/pelvis combination were found (*r* = −0.274, *p* = 0.008, *r* = 0.261, *p* = 0.012, *r* = −0.302, *p* = 0.003; Figure 7). Conversely, no significant correlations between XCOM_ML_ and MRP of both leg coordination, and between XCOM_ML_ and MRP of ipsilateral combination were found (*r* = −0.122, *p* = 0.248, *r* = −0.109, *p* = 0.302, respectively). In addition, no significant correlations between MRP of contralateral combination and MRP of trunk/pelvis combination were found for the 3–4 years, 9–10 years, and young adult groups (*r* = −0.025, *p* = 0.908, *r* = −0.083, *p* = 0.788, *r* = −0.018, *p* = 0.948, respectively; Figure 8). Conversely, significant correlations were found for the 5–6 years (*r* = −0.586, *p* = 0.002) and 7–8 years groups (*r* = −0.522, *p* = 0.046).

## Discussion

This study mainly elucidated the developmental process of inter-limb coordination and trunk twist coordination, and the contribution of inter-limb coordination to dynamic balance control during gait. Mediolateral dynamic stability during gait (MoS_ML_) was not fully mature at age 10. Additionally, mediolateral dynamic balance control (XCOM_ML_) gradually improved with increasing age and was associated with the development of contralateral limb coordination and trunk and pelvic twist coordination. Furthermore, trunk/pelvis coordination correlated with contralateral limb coordination for the 5–6 years and 7–8 years groups, but not the 3–4 years groups, 9–10 years groups, and young adults. These results suggest that relationships between contralateral combination and trunk/pelvis coordination during gait may change from a disorderly coordination strategy in 3–4 years to a tightly linked strategy in 5–8 years. These relationships become altered in children aged 9–10 years and attain an adult-like manner of coordination patterns.

### Maturing Principle of Gait Pattern

No significant between-group differences were found in the upper arm and upper leg angular movements and MRP of both leg combinations in the present study (Table 3; Figure 4). Normalized step parameters (stride length, walking velocity, cadence), kinematics (isolated hip, knee, ankle joint movements), and kinetics (joint moments and joint power) show adult-like patterns by 5 years of age (Sutherland, 1997; Vaughan et al., 2003; Ganley and Powers, 2005; Chester et al., 2006). This suggests that the basic principle of gait pattern might have already been mastered by about 5 years (Hu et al., 2016), which might be because of the acquisition of spinal central pattern generators (CPGs). CPGs are presumably supervised by brain stem structures and the spine, which suggests that CPGs acquisition matures earlier than cortical control (Martin, 2005). Actually, CPGs contribute to the control of the swinging movements of limbs during gait and can be utilized at 2–11 months of age (Lamb and Yang, 2000). Upper arm and leg angular movements and both upper leg coordination might be controlled by CPGs, and thus, take less time to mature, similar to the development of kinematics and kinetics patterns (Figure 3).

### Maturing Trunk and Pelvic Coordination

Although contrary to our hypothesis, no significant between-group differences were found in the MRP of trunk/pelvis coordination (Figure 4E), the trunk angular movement was significantly increased in the 3–4, 5–6, and 7–8 years age groups compared to the young adult group (Table 3), similar to previous studies (Thummerer et al., 2012; Li et al., 2021). Phase coupling between trunk movement and pelvic twist movement is affected more by gait speed than by stride frequency (van Dieën et al., 2021). Normalized walking speed did not differ significantly between groups in this study (Table 3). Thus, trunk/pelvis coordination might not differ between groups. However, each trunk and pelvic twist movement is used to minimize the total body angular momentum (Herr and Popovic, 2008). Furthermore, a greater pelvic movement could facilitate a faster walking speed (Whitcome et al., 2017). Younger children exhibited less mechanical energy-efficient walking (Bach et al., 2021). Therefore, it suggests that greater trunk and pelvic angular displacement in younger children might be controlled to create higher energy and faster walking speed. The present study indicated that no significant difference between young adults and 3–10 years children in phase coupling movement between the trunk and pelvic twist movement was found, but each range of motion in trunk and pelvis attained adult-like level at 7–8 years of age.

### Development of Inter-limb Coordination

Although, MRP of both arm combination and ipsilateral combination significantly increased in the young adult group compared to the 3–4 years age group (Figures 4A,C), MRP of contralateral coordination significantly increased in the young adult group compared to 3–4, 5–6, and 7–8 years age groups (Figure 4D). These findings suggest that contralateral inter-limb coordination takes longer to mature than ipsilateral inter-limb coordination. A higher order regulation of interlimb coordination can be achieved at the brainstem and cortical level (Debaere et al., 2001). Moreover, even for the natural swinging of the arms, it has been shown that cortical contributions may be present (Barthelemy and Nielsen, 2010). Thus, inter-limb coordination, including arm swing (ipsilateral combination) takes longer to mature than both upper leg combinations. Furthermore, the results of the present study indicated that contralateral coordination gradually changed to an in-phase pattern with increasing age and took longer to mature than ipsilateral coordination (Figure 4D; Meyns et al., 2013, 2020). Contralateral limb coordination is accomplished by control of both factors including bilateral and arm and leg. Bilateral movement becomes less automated and requires greater sensorimotor cortical input (Richmond and Fling, 2019). The corpus callosum mediates the transfer and integration of lateralized cognitive, motor, and sensory information between cortices (Richmond and Fling, 2019). Even in teenagers, the maturation of transcallosal pathways is ongoing (Ciechanski et al., 2017). Therefore, the present results found that contralateral limb coordination may have required a greater control process, and thus better coordination, defined as closer to in-phase coordination and reached an adult-like level at 9–10 years of age (Figure 4D).

Contralateral limb coordination depended on trunk/pelvis coordination in the 5–6 years and 7–8 years groups (Figure 8). This result indicates that a different control strategy was used between children 3–4 years of age and 5–8 years of age, and between children 5–8 years of age and over 9 years of age. The arm movements during gait were adjusted by cortical control (Barthelemy and Nielsen, 2010). Children at age 3–8 years depend on “en bloc” postural strategy (Assaiante, 1998; Sveistrup et al., 2008). Thus, the CNS may prioritize controlling trunk and pelvic movements and select the “en bloc” strategy to adjust arm swing. However, no significant correlations between MRP of contralateral combination and trunk/pelvis coordination were found in the 3–4 years group (Figure 7). More variable relationships in the 3–4 years group were observed (Figure 8). Thus, it suggests that the 3–4 years group showed an immature and disorderly coordination strategy. Conversely, no significant correlations between MRP of contralateral combination and MRP of trunk/pelvis coordination were found in the 9–10 years and young adult groups (Figure 8). There is a task-dependent switch from direct cortical-motoneuronal control during skilled hand movements to indirect control by cervical propriospinal circuits during locomotion (Dietz, 2010; Meyns et al., 2020). Limb swing and trunk movements may be controlled independently to utilize limb swing effectively and flexibility according to task contexts. Coordination control strategy during gait may become altered in children aged 9–10 years and attain adult-like coordination patterns.

### Development of Dynamic Balance Control

The dynamic balance control (XCOM_ML_) gradually improved (Figure 6), but the dynamic postural stability (MoS_ML_) was not fully matured until age 10 or older (Figure 5). Furthermore, XCOM in the mediolateral axis was linked with both upper arm coordination, contralateral limb coordination, and trunk and pelvic twist coordination (Figure 7). That is, closer to the anti-phase pattern of both upper arm coordination and closer to the in-phase pattern of contralateral limb coordination decreased the XCOM_ML_. Conversely, the out-of-phase pattern (about 90 degrees) of trunk/pelvis coordination increased the XCOM_ML_. The results of our study support previous studies (Hallemans et al., 2018; Sidiropoulos et al., 2021). Appropriate arm swing and posture of the arms during gait will optimize stability (Meyns et al., 2014), and minimize energy consumption by decreasing angular momentum around the vertical axis (Park, 2008). Furthermore, both the pelvis and thorax contributed to decreasing total body angular momentum and limb swing (Bruijn et al., 2008). Therefore, the results of our study indicated that it was important for dynamic balance control to develop adequate inter-limb coordination and trunk and pelvic twist coordination. Although contralateral limb coordination attained adult-like levels by age 10, the dynamic postural stability continued to improve after age 10 (Figure 5), suggesting there are other factors influencing the maturation of dynamic postural stability during gait, such as muscle synergy (Bach et al., 2021) and/or neural networking (Corporaal et al., 2018). In future studies, electromyography and electroencephalography should be performed to analyze relationships among inter-limb coordination and dynamic postural stability.

### Limitations

There are several limitations to this study. The anthropometric model used from Jensen’s report (Jensen, 1986) was developed with a population of male children aged 4–15 years. The present study population included approximately 54% females and children as young as 3 years of age, which may have influenced the XCOM and COM velocity results. Furthermore, there are various differences between boys and girls in gait control (Di Nardo et al., 2017; Whitcome et al., 2017), but sex-related variations could not be detected in this study. Therefore, future investigations into the sex differences associated with the development of inter-limb coordination during gait will likely yield valuable insights. The markers on the lower limbs were placed on the skin or the light underwear, which might create measurement errors associated with the relative movement between the markers and the underlying body segments owing to the movement of the cloth. Trunk angular movement was defined as the angle between the mediolateral axis and a line along both acromioclavicular joints in a transverse plane. The acromioclavicular joints move significantly with respect to the trunk during arm swing. Thus, the effects of arm swing on the trunk axial rotation might be included. Although the forearm and lower leg affect the gait patterns, inter-limb coordination is only limited to upper arms and upper legs in this study. Thus, in future studies, coordination of the forearm and lower leg should be analyzed. Finally, in this study, walking speed did not differ significantly between groups (Table 3). However, the velocity of the COM incorporated into the XCOM was not normalized to √*gl* based on the previous study (Hof et al., 2005). Therefore, the effects of walking speed on the XCOM might not be excluded.

## Conclusion

This study demonstrated the development of dynamic postural stability and balance control with inter-limb coordination and trunk and pelvic twist coordination during gait. Dynamic postural stability does not fully mature until age 10 or older. Improving dynamic balance control is associated with the development of in-phase pattern of the contralateral upper arm and upper leg coordination, and the anti-phase pattern of both upper arm coordination. Furthermore, the out-of-phase pattern (about 90 degrees) of the trunk and pelvic twist coordination is also negatively correlated with dynamic balance control. Finally, contralateral upper arm and upper leg coordination is linked with trunk and pelvic twist coordination at 5–8 years of age.

## Data Availability Statement

The raw data supporting the conclusions of this article will be made available by the authors, without undue reservation.

## Ethics Statement

The studies involving human participants were reviewed and approved by Ethical committee of Oita University Faculty of Welfare and Health Science and Sapporo Medical University Ethics committee. Written informed consent to participate in this study was provided by the participants’ legal guardian/next of kin. Written informed consent was obtained from the individuals, and minors’ legal guardian/next of kin, for the publication of any potentially identifiable images or data included in this article.

## Author Contributions

HM: conception, investigation, methodology of this study, data analysis, drafting and revising the manuscript. SM, NK, TI, and NH: conception, investigation, methodology of this study, and editing the manuscript. TA: conception and supervision of the study and editing and revising the manuscript. All authors contributed to the article and approved the submitted version.

## Conflict of Interest

The authors declare that the research was conducted in the absence of any commercial or financial relationships that could be construed as a potential conflict of interest.

## Publisher’s Note

All claims expressed in this article are solely those of the authors and do not necessarily represent those of their affiliated organizations, or those of the publisher, the editors and the reviewers. Any product that may be evaluated in this article, or claim that may be made by its manufacturer, is not guaranteed or endorsed by the publisher.
